# Failing Heart Transplants and Rejection—A Cellular Perspective

**DOI:** 10.3390/jcdd8120180

**Published:** 2021-12-12

**Authors:** Maria Hurskainen, Olli Ainasoja, Karl B. Lemström

**Affiliations:** 1 Division of Pediatric Cardiology, New Children’s Hospital, Helsinki University Hospital and University of Helsinki, 00290 Helsinki, Finland; 2 Pediatric Research Centre, New Children’s Hospital, Helsinki University Hospital and University of Helsinki, 00290 Helsinki, Finland; 3 Transplantation Laboratory, Helsinki University Hospital and University of Helsinki, 00014 Helsinki, Finland; olli.ainasoja@helsinki.fi (O.A.); karl.lemstrom@helsinki.fi (K.B.L.); 4 Department of Cardiothoracic Surgery, Helsinki University Hospital and University of Helsinki, 00290 Helsinki, Finland; 5 Translational Immunology Program Research Programs Unit, University of Helsinki, 00290 Helsinki, Finland

**Keywords:** heart transplantation, ischemia reperfusion injury, acute rejection, cardiac allograft vasculopathy, cellular disease mechanism

## Abstract

The median survival of patients with heart transplants is relatively limited, implying one of the most relevant questions in the field—how to expand the lifespan of a heart allograft? Despite optimal transplantation conditions, we do not anticipate a rise in long-term patient survival in near future. In order to develop novel strategies for patient monitoring and specific therapies, it is critical to understand the underlying pathological mechanisms at cellular and molecular levels. These events are driven by innate immune response and allorecognition driven inflammation, which controls both tissue damage and repair in a spatiotemporal context. In addition to immune cells, also structural cells of the heart participate in this process. Novel single cell methods have opened new avenues for understanding the dynamics driving the events leading to allograft failure. Here, we review current knowledge on the cellular composition of a normal heart, and cellular mechanisms of ischemia-reperfusion injury (IRI), acute rejection and cardiac allograft vasculopathy (CAV) in the transplanted hearts. We highlight gaps in current knowledge and suggest future directions, in order to improve cellular and molecular understanding of failing heart allografts.

## 1. Heart Transplantation—No Improvements in Long Term Survival

Heart transplantation is the only treatment option for selected patients with heart failure. Worldwide, over 5000 heart transplantations are performed annually. With developments in immunosuppressive care, recipient and donor selection and heart preservation methods, the short-term survival of the patients has increased significantly [1]. However, the long-term survival of heart transplant patients remains limited [2]. Between 2002–2009 the median post-transplant survival was 12.5 years, and improved to 14.8 years among one-year survivors. Survival was improved by factors such as cardiomyopathy as the primary diagnosis, younger age of the recipient and female sex [2]. In addition to limited survival, heavy immunosuppressive medication together with intensive clinical follow-up, place a significant burden to the quality of life of the patients. To maximize the lifespan of heart transplants, it is necessary to understand the cellular and molecular mechanisms of the allograft failure in detail. This knowledge will enable innovation of targeted therapies and create precision assays for monitoring the disease processes in the heart tissue.

Despite decades of investigation, significant gaps in knowledge remain in understanding how the structural cells of the heart participate in the immune response, and how the injury and repair mechanisms of the allograft are controlled. The structural cells of the heart are known to contribute to the immune response [3] and will determine whether the tissue will eventually scar or develop vasculopathy. Thus, it is critical to gain a better understanding of the temporal and spatial cellular mechanisms of all pathological processes affecting the allograft, in order to discover the core mechanisms determining the fate of the tissue. Eventually, the goal would be to pharmacologically not only to suppress the immune cells with immunosuppressive medication, but also to control the immune responses in the structural cells and promote their reparative signals.

The novel single cell methods have opened a new era for studying complex pathological processes in tissues, leading us to a new level of understanding the cell-specific signaling driving disease processes [4,5,6,7]. Single cell transcriptomic analysis aims to describe cellular functions in homeostasis and in disease, from the level of genes to the level of cellulomes and connectomic networks within tissues and, thus, provides important and critical steps in understanding cellular functions on the tissue level. Although transplant-related single cell studies are not yet available, studies in the normal heart, animal models of heart disease and in patient samples have already resulted in the construction of a novel heart cell atlas, and provided new insights into disease mechanisms [8,9]. Moreover, single cell multiomics integrating different single cell modalities holds great promises for understanding heart allograft failure in unprecedented levels [10].

Here, we will review the normal cellular composition of the heart in the light of novel single cell research, as well as the cellular mechanisms of some of the major pathological processes in the heart allograft including ischemia reperfusion injury (IRI), acute rejection, and cardiac allograft vasculopathy (CAV).

## 2. The Normal Cellular Composition of the Heart

The heart is anatomically and functionally complex and requires delicate orchestration of several cell populations to enable controlled contraction and relaxation. Recent advancements in the robust and high-throughput single cell technologies have significantly contributed to our understanding of the cellular complexity of the heart [8]. An extensive intercellular communication network between cardiomyocytes (CMs) and non-cardiomyocyte cells of the heart maintain the complex and coordinated physiological functions of the heart.

### 2.1. Cardiomyocytes

CMs are joined together in sheets by intercalated discs and arranged in helical spiral pattern forming a thick layer between the epicardium and the endocardium of the heart. Cardiomyocyte fibers are surrounded by a dense capillary network—no cardiac myocyte is more than 2–3 µm away from a cardiac microvascular endothelial cell (EC). CMs are responsible for cardiac contraction, regulated by the cardiac conduction system coordinating the depolarization of electrically coupled CMs. Single cell studies in human heart suggest the presence of many different types of CMs and a markedly distinct transcriptional signature for atrial (aCM) and ventricular CMs (vCM) [11,12]. Both studies report altogether five different vCM populations, some of these distinct to the right or left ventricle with transcriptional signature indicating differing functionality. In addition, five different aCM populations are reported. Similar to vCM populations, the different aCMs have distinct transcriptional features suggesting differences in origin and functionality [12]. Transcriptional differences between aCM and vCM populations indicate different developmental origins, hemodynamic forces and specialized functions in cardiac chambers.

### 2.2. Endothelial Cells

The inner layer of the heart chambers, the endocardium, is lined with specialized ECs, which during embryogenesis give rise to the endothelial lining in the heart vasculature. Endothelium regulates vascular health by controlling vascular tone, aggregation of platelets, and adhesion of circulating leukocytes [13,14]. Heart vasculature consists of large epicardial coronary arteries (>500 µm), which divide into prearterioles (100–500 µm), intramyocardial arterioles (<100 µm), and capillaries. Cardiac oxygen consumption and supply occurs on the level of intramyocardial arterioles and capillaries, making these vessels most vulnerable. Single cell studies in human heart tissue have found several distinct EC populations of capillary, arterial, venous and lymphatic subtypes, lining different types of vessels and with distinct transcriptomes and functions [11,12].

### 2.3. Stromal Cells

#### 2.3.1. Fibroblasts

Fibroblasts (FBs) are found in the cardiac skeleton, where they form the valvular components and connect them to the septa, and within the myocardial interstitium, where they are closely connected to other cell types [15]. Most fibroblasts are originated from the epicardium [16]. FBs are the major cell type responsible for creating and maintaining the extracellular matrix (ECM) surrounding the cardiomyocyte. The ECM substances such as collagen, elastin and glycosaminoglycans create elasticity in the cardiac muscle. Cardiac fibroblasts also communicate with immune cells, sustain CM electrical coupling and sense stress [8]. Single cell studies of human heart tissue have recognized a previously underestimated heterogeneity in FB populations [12]. Litviñuková et al. identified seven distinct FB populations with regional enrichment in ventricles or atria, with distinct functionalities such as stronger profibrotic responses in atrial FB. This heterogeneity is in concordance with the murine heart single cell studies [17,18,19]. The cellular diversity of FBs is thought to be necessary to ensure that CMs can be supported by FBs in response to varying biophysical stimuli.

#### 2.3.2. Other Stromal Cells

Pericytes and vascular SMCs stabilize vessels through interactions with adjacent endothelial cells and have contractile functions to regulate the blood flow [14,20]. During embryogenesis, endocardial ECs differentiate into both of these cell types [21]. Pericytes are located around microvascular blood vessels, whereas SMCs cover larger arterioles, arteries and veins. A single cell study in human heart identifies four distinct clusters of pericytes, with a distinct location to atria or ventricles [12]. One of the clusters presents as a transitioning pericyte to endothelial cell type. Vascular SMCs are split into two types, one of them expressing stem cell markers and suggested to derive from veins, whereas the other is suggested to derive from arteries.

### 2.4. Immune Cells

The immune system contributes to cardiac development, composition and function—the immune cells infiltrate the heart at gestation and remain in the myocardium, where they participate in essential housekeeping functions throughout life [22]. Heart resident immune cells include both myeloid and lymphoid cell populations [23]. Macrophages are the main resident immune cells of the heart and are responsible for phagocytosing bacteria and apoptotic cells, but have also important regenerative roles [24]. Dendritic cells (DCs) are important in activating the adaptive immune system by antigen presentation. Mast cells are thought to be early triggers of immune responses. Additionally, a small number of resident B and regulatory T cell subsets are found in the heart. In the so far largest human heart single cell study, 21 different immune cell populations were identified. These include ten macrophage groups, two groups of NK cells, dendritic cells, two groups of both CD8+ T cells and CD4+ T cells, as well as a group of mast and plasma cells [12].

### 2.5. Neurons

Neuronal cells are also found in the heart. In addition to neuronal control, neurons have immunoregulatory properties in many tissues, although studies in the heart remain elusive [25,26]. In addition, the role of neurohumoral cardiac regulation is emerging [27].

## 3. Ischemia-Reperfusion Injury—Early Event, Long-Term Consequences

**Figure 1:**
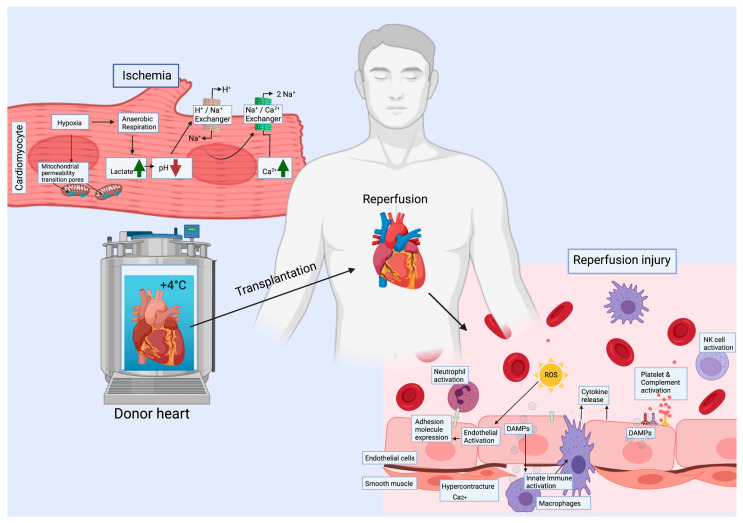
Ischemia and reperfusion induce many cellular changes. During ischemia, all cell types are hypoxic and shift to anaerobic metabolism accompanied with a decrease in pH. As a consequence, the level of Ca^2+^ increases. During reperfusion, release of ROS and DAMPs will lead to immune activation with cytokine release, expression of adhesion molecules in endothelial cells and to recruitment of immune cells, as well as platelet and complement activation. The metabolic switch to aerobic accompanied by high intracellular Ca^2+^ may cause hypercontracture of the cardiac muscle. ROS = reactive oxygen species, DAMPs = damage associated molecular patterns.

Significant steps determining the functionality of the allograft take place already before transplantation, including donor brain death [28]. One of the most important factors is heart allograft preservation, the standard procedures involving cold preservation and perfusion with storage solution. The ischemia period should not exceed four hours due to irreversible muscle damage caused by ischemia [29]. Reperfusion of the allograft will initiate a complex interplay of pathological processes, the degree of which will determine the amount of immediate muscle damage, and predispose the heart to further pathological processes such as acute rejection [30] and development of cardiac allograft vasculopathy (CAV). Ischemia reperfusion injury (IRI) involves a complex coordinated interplay of fibroblasts, leukocytes, endothelial cells, pericytes, and cardiomyocytes [31] (Figure 1). In myocyte damage, the process will lead to the replacement of tissue by scarring. Results from single cell studies have revealed an underappreciated diversity of cellular events occurring during the process of IRI in acute myocardial infarction (MI) [18,19,32,33,34,35,36,37], but the detailed cellular events are not so well described in transplant related IRI, which has some distinct cellular mechanisms, as it presents as global transient ischemia accompanied by hypoxic regions.

### 3.1. Cardiomyocytes (CMs)

Myocyte death is the ultimate result of prolonged ischemia, and extended myocyte damage will lead to heart failure. During ischemia, the metabolism of CMs slows down significantly and switches from aerobic to anaerobic, causing slow depletion of ATP and accumulation of mitochondrial metabolic byproducts, such as free oxygen radicals. Eventually, ischemia causes cellular swelling, lactic acidosis and accumulation of H^+^ [38]. The excess H^+^ is removed via Na^+^/K^+^ pump, resulting in consequent accumulation of Na^+^ followed by Ca^2+^, via activation of the Na^+^/Ca^2+^ pump. Immediately after reperfusion, high reactive oxygen species (ROS) formation is observed. ROS together with the high Ca^2+^ level leads to opening of the mitochondrial permeability transition pore (mPTP) and release of cytochrome c into the cytoplasm, inducing a cascade of apoptosis. Mitochondria and unregulated opening of mPTP are key factors in IRI and heart failure [39]. Furthermore, the decreased ATP causes myofibrillar shortening and moderate contracture with cytoskeletal changes, making the cells fragile. Reperfusion may lead to hypercontracture causing rise in end-diastolic pressure and ventricular wall stiffness [38].

In addition to increased ROS, CM death will also lead to release of cellular components into the extracellular space and the circulation, acting as damage associated molecular patterns (DAMPs), which can be recognized by pattern recognition receptors (PRRs) on the surface of immune and some structural cells such as ECs [40,41]. Activation of these receptors will induce the nuclear translocation of transcription factors such as NF- κβ, with consequent expression of the downstream proinflammatory target genes leading to the release of cytokines.

A recent single cell study of cardiac ischemia in a murine MI model shows that DNA-binding transcription factor ZEB2 is induced in cardiomyocytes as a result of ischemia, leading to secretion of factors involved in cardiac remodeling, such as circulating TMSB4 and PTMA. These have been linked to cardioprotection via neovascularization, angiogenesis and apoptosis [37]. Another single cell study shows that a subset of CMs activates the wound healing response in FBs in a paracrine manner via secreting elevated levels of beta-2 microglobulin in response to ischemic damage [33].

### 3.2. Endothelial Cells (ECs)

**Figure 2:**
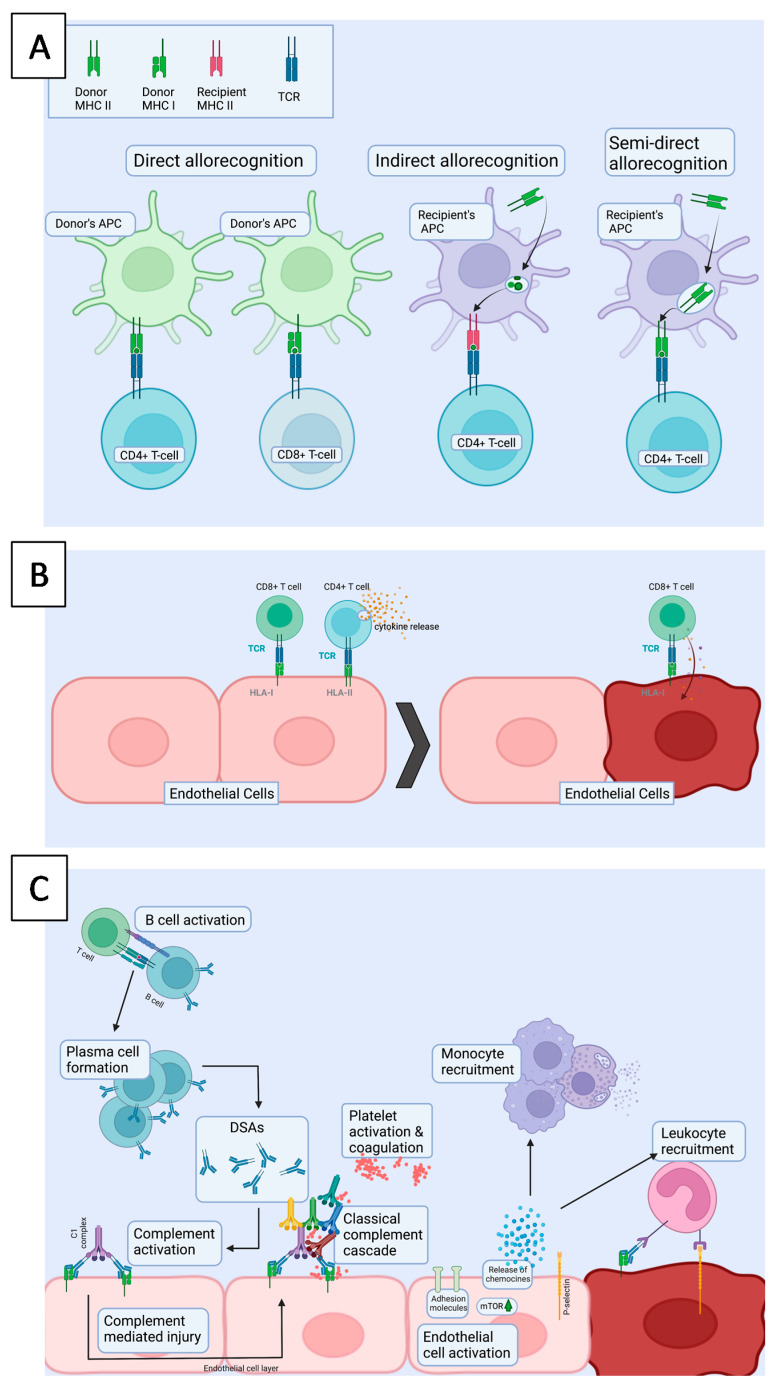
(**A**). Different T cell allorecognition pathways. In direct allorecognition, donor derived APCs present donor allopeptides on a donor MHC to the recipient’s T-cells, which leads to donor allorecognition. In indirect allorecognition, recipient derived APCs present a donor allopeptide on MHC molecule to the recipient’s T-cell. In semi-direct allorecognition, recipient APC catches a donor MHC molecule, which is transported to the cell surface and presented to T-cells. (**B**). In cellular rejection, alloreactive cytotoxic CD8+ T cells have been activated in secondary lymphoid organs by activated antigen presenting cells either via direct or indirect allorecognition. Once they encounter cells presenting target antigens on HLA I molecule, the target cells, which are typically ECs, will be killed. (**C**). AMR is characterized by injury of the allograft endothelium and presents as microvascular inflammation. First, donor derived antigen is presented by APCs to CD4+ T-cells in the secondary lymphoid organ. Hence, CD4+ T cells activate B cells and the formation of plasma cells, producing donor specific antibodies (DSAs). Upon DSA (IgG) binding to target cells, which are typically ECs, the activation of complement cascade is triggered, leading to the activation of membrane attack complex. HLA binding activates intracellular signaling in ECs, e.g., via mTOR, which induces upregulation of adhesion molecules and further leukocyte recruitment. APC = Antigen presenting cell, TCR = T cell receptor, MHC = major histocompatibility complex, mTOR = mammalian target of rapamycin.

IRI promotes vascular endothelial dysfunction leading to inflammatory activation, coagulation and vasomotor disturbances in a region-specific manner. Ischemia promotes acidosis in ECs followed by increased intracellular Ca^2+^ from the endoplasmic reticulum (ER), and consequent initiation of apoptosis [42]. With reperfusion, production of ROS and release of DAMPs from dying cells will activate the inflammatory, hypercoagulatory, and vasoactive pathways in ECs. Upon reperfusion, ECs upregulate the expression of leukocyte adhesion molecules such as selectins and ICAMs in order to recruit neutrophils. As a part of the immune response, the complement system is activated. Disruption of the endothelial barrier integrity allows leukocyte infiltration into the interstitium [43]. The inflammatory activation may result in the presentation of alloantigens and trigger acute rejection (Figure 2).

In addition to immune activation, EC vasoconstriction is promoted by upregulation of endothelin-1 expression and downregulation of nitric oxide (NO) expression. The simultaneous vasoconstriction, upregulation of procoagulant genes and platelet activation create a prothrombotic environment [31].

Endothelial dysfunction may lead to uneven myocardial perfusion, causing further hypoxia in affected regions. Hypoxia, in turn, has been shown to induce phenotypic changes consistent with endothelial-to-mesenchymal transition (EndMT) [44,45], where ECs undergo phenotypic changes and transdifferentiate into myofibroblast-like cells with increased production of extracellular matrix proteins contributing to fibrosis.

In a single cell study of cardiac ischemia caused by myocardial infarction (MI), ECs undergo transient mesenchymal activation (EndMA) that is associated with profound metabolic adaptations [34]. The mesenchymal marker expression is no longer elevated ten days post MI, suggesting that EndMA likely represents a reversible continuum in response to a hypoxic and inflammatory injury environment, instead of a differentiation process in its classical sense. This partial EndMA is thought to contribute to new vessel growth, by promoting a pro-migratory and pro-invasive state, and suggests that the contribution of EndMA to cardiac fibrosis is limited. A study of murine MI characterizes the different endothelial cell contributions to ischemia response, neovascularization and tissue regeneration, and suggest that the main mechanism of neovascularization takes place via clonal expansion [35]. Furthermore, the study identifies a link between *Plvap* expression and the EC proliferation capacity necessary to promote cardiac regeneration after ischemia.

### 3.3. Stromal Cells

#### 3.3.1. Fibroblasts and Myofibroblasts

FBs contribute to heart repair and remodeling, immune cell recruitment, and fibrotic scar formation [46]. Hypoperfusion-induced hypoxia caused by IRI, as well as the presence of DAMPS, are potent profibrogenic stimuli for the cardiac FBs. Prolonged ischemia will result in loss of CMs and induce activation of FBs, required for proper scar formation. Myofibroblasts are activated fibroblasts found in hypoxic regions with some ability to contract as a property of smooth muscle cells [47]. TGFβ and changes in the biochemical properties of the cardiac muscle promote the activation of fibroblasts, which are characterized by the expression of genes that encode contractile proteins such as Acta2 and Tagln, and ECM components such as PostnF, and Col1a1 [46]. Activated FBs will proliferate and have altered ECM metabolism, leading to accumulation of ECM proteins due to increased matrix synthesis and decreased expression of matrix metalloproteases, responsible for the degradation of ECM. Activated FBs will also stimulate ECs in order to promote angiogenesis and revascularization via secretion of Angpt1 [48] and VEGF [49]. Activated FBs contribute to the immune response by secreting hematopoietic growth factors such as GM-CSF [50].

In single cell studies of ischemia in a murine MI model, specific stromal cell populations show temporal activation. Early transition of a FB subtype to myofibroblasts seems to be an important step determining reparative outcome [36]. Another study shows a new reparative subpopulation of fibroblasts expressing *Cthrc1* in a murine MI model, and the presence of a similar population can also be seen in a swine model and in human patients [19]. Furthermore, Farbehi et al. show that a subpopulation of myofibroblasts supports anti-fibrotic programs [18].

#### 3.3.2. Pericytes

Pericytes have a key role in regulating capillary blood flow by contracting and dilating. In brain ischemia, peroxynitrite causes pericyte contraction and capillary constriction [51]. The same phenomenon has been shown in coronary capillaries where the microvascular blood flow is reduced after ischemia due to pericyte constriction leading to the no-reflow phenomenon [52]. The pericyte-induced cellular communication in IRI is not well characterized.

### 3.4. Immune Cells

Along with reperfusion, the ROS and DAMPs released in the ischemic heart tissue initiate a sterile inflammatory response. Cytokine and chemokine production by endothelial cells and tissue resident immune cells (macrophages) will lead to the activation of an innate immune response first by recruitment of proliferating neutrophils to the site of injury. This is followed by further secretion of cytokines and chemokines by neutrophils, in order to recruit additional immune cells, such as NK cells and monocytes, which can differentiate into macrophages and dendritic cells (DCs) [53]. Recipient NK cells are suggested to undergo priming to full effectors upon IRI, which may have long-term consequences in later vulnerability to rejection episodes [54]. Presentation of alloantigens by antigen presenting cells will induce an adaptive immune response and T cell allorecognition, leading to prolonged inflammation.

At first, innate immune cells scavenge dead material, and scavenger receptors such as MERTK are activated. Cells will release proinflammatory cytokines such as IL-1, TNF and IL-6. Over the course of several days, the inflammatory phase gives way to a reparative phase, which is dominated by the disappearance of neutrophils and the appearance of Ly6C low macrophages. Neutrophils may have reparative functions via macrophage M2 polarization [55]. The mechanisms by which antigen-specific T cells are activated during sterile inflammation are not well understood [30]. During the reparative phase, due to Treg produced IL-10 [56] and intrinsic signals such as NR4A1 [57], the production of inflammatory cytokines, growth factors and chemokines, decreases. Mast cells accumulate in the heart, and they are believed to have important preserving functions for cardiac contractility via myofilament phosphorylation.

Recent single cell studies have provided further insights into the immune cell-related mechanisms of ischemia. In a single cell ischemia study of MI, a resident cardiac macrophage population was found to protect the heart from adverse remodeling in the infarct zone [32]. Single cell studies have also revealed a functionally unique heart regulatory T cell (Treg) population that was found to be cardioprotective in a murine heart ischemia study of MI [58]. In particular, the Sparc expression in Tregs acts as a critical factor in protecting the heart against MI, by increasing collagen content and boosting maturation in the infarct zone.

## 4. Acute Rejection—Better Immunosuppression, Declining Morbidity

An allogenic heart transplant is non-self to the recipient and prone to allograft rejection by the recipient’s immune system. With improved immunosuppressive medication, acute rejection episodes have declined, but are still one of the major causes of death among heart transplant patients. Acute rejection follows allorecognition (Figure 2A) and involves different mechanisms including cellular (Figure 2B) and antibody mediated (AMR) rejection (Figure 2C). Both types of rejection have distinctive histological and immunohistochemical findings and the golden diagnostic standard of rejection is EMB.

### 4.1. Cellular Rejection

In direct allorecognition (Figure 2C), the activated donor APCs migrate to secondary lymphoid tissue and present donor peptides with either MHCI or MHCII receptors to CD8+/CD4+ naïve T cells, resulting in the development of alloreactive effector T cells [59,60]. CD8+ T cells act via proinflammatory cytokine production and direct destruction of allogenic tissue. CD4+ Th1-type cells and macrophages are responsible for delayed hypersensitivity and inflammation. The consequent cellular rejection is histologically characterized by diffuse lymphocytic infiltrates comprising mainly of T cells and macrophages, myocyte damage and in severe cases, edema, hemorrhage and vasculitis in the endomyocardial biopsies.

### 4.2. Antibody-Mediated Rejection

AMR begins with indirect presentation (Figure 2C) of a foreign (donor-derived) antigen by recipient antigen presenting cell (APC) to CD4+ T cells, which activate B cells and trigger the formation of plasma cells capable of producing donor-specific antibodies (DSA). The anatomical site(s) of B cell differentiation to plasma cells and DSA production are unknown [61]. Upon DSA binding to their specific antigens on the cell surface, complement cascade activation is initiated, leading to cell death. As all DSA are not equally pathogenic, AMR is identified from endomyocardial biopsies by distinct microvascular histological lesions including capillary injury, positive staining for CD68 and C4d in endomyocardial biopsies. The diagnosis may be accompanied by the presence of DSAs [62,63].

#### 4.2.1. Endothelial Cells

Endothelium forms a physical barrier between the donor organ and the recipient as the primary target of alloresponses. Endothelium is prone to inflammation, alloreactive lymphocytes, donor-specific antibodies and complement activation. As a consequence, ECs in allotransplants are targets of AMR, especially as they are capable of expressing HLA molecules, exposing the endothelium to allorecognition. The endothelium participates in vascular and immune homeostasis, and actively participates in cross-talk with the surrounding cells, driving many of the consequent changes in acute rejection [64]. ECs have intricate mechanisms of participating in the immune response. On the presence of TNFα and Il1α (proinflammatory cytokines), the costimulatory molecule ICOSL is strongly expressed by endothelial cells, allowing CD4+ T cell activation [65]. Other cytokines such as IL2 and IFNγ have been shown to allow ECs expressing HLA-DR to activate CD+ T cell differentiation towards the Th17 and Treg subsets [66]. Alloantibody binding to endothelial HLA class I or II molecules mediates its effects on ECs via signal transduction, and the mechanisms for HLA I or II are distinct [67]. Activated ECs promote further inflammation by upregulating adhesion molecules and recruiting leukocytes via cytokine and chemokine release [64].

#### 4.2.2. NK Cells

Several studies have implied the importance of NK cells in initiating allorecognition [64,68]. DSAs binding to endothelial cells may lead to the ligation of Fc receptors on NK cells and contribute to antibody-mediated cytotoxicity or to the release of proinflammatory cytokines, such as IFNγ. This, in turn, may increase the HLA expression on the cell surface and propagate antibody-mediated damage. In addition, NK cells seem to be capable of allorecognition by sensing the absence of self HLA class I molecules [54].

#### 4.2.3. Monocytes and Macrophages

Macrophages are capable of antigen processing and presentation, co-stimulation, cytokine production and tissue remodeling, and are thus being important mediators of rejection [69]. The majority of leukocytes found in endothelial infiltrates in AMR represent CD68+ monocytes/macrophages. The expression of the adhesion molecule P-selectin on ECs increases monocyte adhesion and recruitment to endothelium [70]. AMR induces upregulation of the Notch ligand Dll4 on ECs and macrophages, inducing the differentiation of monocytes towards proinflammatory M1 type macrophages observed in endomyocardial biopsies [71].

#### 4.2.4. Cardiomyocytes

Myocyte damage is a feature of cellular rejection. In addition to T cell induced CM necrosis close to the lymphocytic infiltrates, also CM apoptosis occurs frequently. Apoptotic CMs are distributed within and remote from the foci of lymphocytic infiltrates. Inflammatory cytokines secreted either from the foci of lymphocytic infiltrate or alternatively, from diffuse macrophage infiltrations are thought to initiate apoptosis [72].

## 5. Cardiac Allograft Vasculopathy—Endothelium as a Key Player

**Figure 3:**
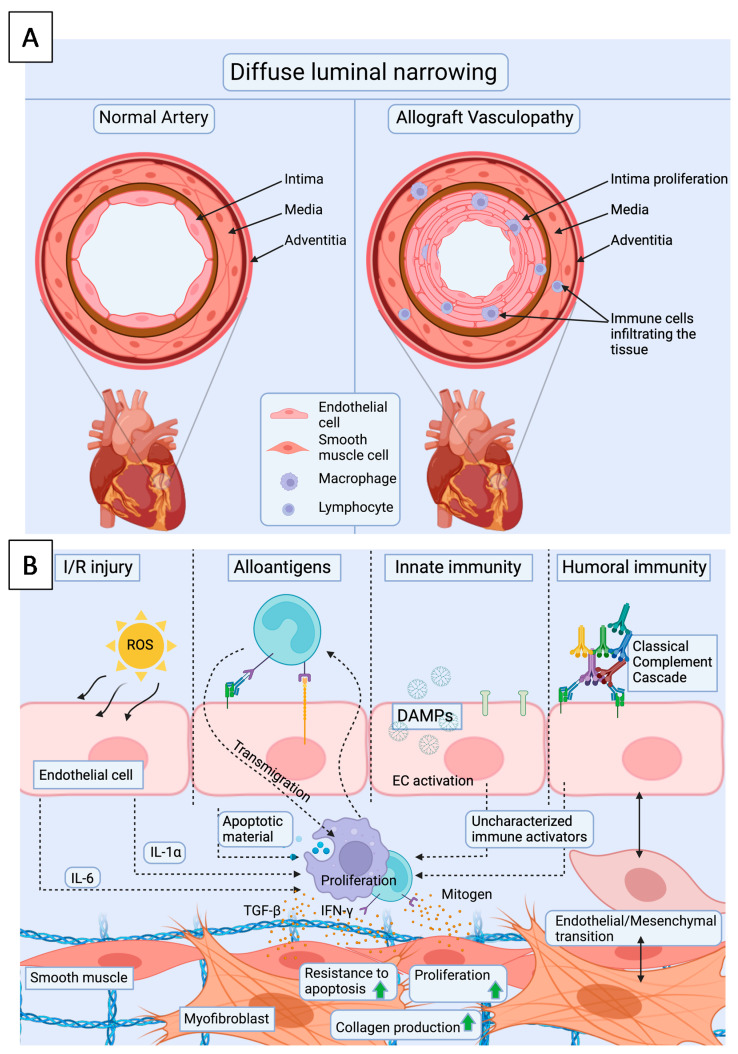
(**A**). Cardiac allograft vasculopathy presents as diffuse intimal thickening of coronary arteries and microvasculature, which is also accompanied by infiltration of immune cells. (**B**). Repetitive EC injuries by ischemia reperfusion injury and rejection episodes initiate the development of CAV. Upon these injuries, ECs upregulate adhesion molecules and recruit leukocytes. Consequent upregulation of cytokines and growth factors will lead to migration and/or proliferation of intimal SMCs as well as endothelial mesenchymal transition, increased resistance to apoptosis, increased ECM production and fibrosis. I/R injury = ischemia reperfusion injury, DAMPs = damage associated molecular patterns, ROS = reactive oxygen species.

One of the most important conditions limiting the long-term survival of heart transplant is cardiac allograft vasculopathy (CAV), which is a distinct form of coronary disease [73,74]. The condition may initiate early during the first year after transplantation [75,76], and due to transplant denervation, progress silently to ventricular arrhythmias and heart failure. In CAV, endothelial dysfunction leads to pathological changes in coronary arteries and in the intramyocardial microvasculature, which undergo diffuse intimal proliferation resulting in luminal stenosis, small vessel occlusion and intravascular hemorrhage [77] (Figure 3A). Intimal thickening is a result of smooth muscle cell (SMC) proliferation and ECM formation, but the intima also contains angiogenic microvessels, infiltrates of macrophages and T cells. In tunica adventitia, B cell, T cell and myeloid cell aggregates are found, whereas the tunica media seems normal [78]. The duration and number of acute rejection episodes are independent risk factors for CAV [79]. In particular, AMR predisposes to the development of early and more severe CAV [80]. Furthermore, the presence of DSAs against endothelium or HLA, increases the risk of CAV even independently of AMR [79]. In addition to alloreactivity, also autoantibodies against cardiac myosin [81] and vimentin [82] may increase the risk of CAV. Donor brain death [83] and CMV infection [84,85] are known to predispose the heart to the development of CAV. Additionally, common risk factors of atherosclerotic coronary disease, such dyslipidemia, obesity, tobacco use and diabetes are risk factors for the development of CAV [86].

### 5.1. Endothelial Cells

The repetitive endothelial injuries caused by IRI and later, by rejection episodes, are thought to be the initial triggers leading to vascular remodeling and SMC proliferation seen in CAV (Figure 3B) [87]. The mechanisms of endothelial activation with a focus on microvascular activation in IRI and acute rejection have been reviewed in previous sections. In CAV, the site of injury is the whole arterial vasculature of the transplant starting from the larger coronary arteries, and extending to the microvasculature. The re-endothelialization of tunica intima after injury may result in chimeric presence of donor- and recipient derived cells [73,88,89,90,91,92,93]. The origin of the recipient-derived neointimal cells has been suggested to derive either from the bone-marrow, the circulating progenitor cells, or from the vascular bed. Endothelial cell replacement is mostly present in the small epicardial and intramyocardial vessels, which are first affected by CAV [94]. This may contribute to altered endothelial phenotypes, which predispose the intima to alloresponse and following CAV. In addition, AMR-associated inflammation, alloantibodies, and activation of the complement cascade may alter the endothelial phenotypes.

### 5.2. Smooth Muscle Cells

The migration and proliferation of SMCs into the intimal region of the vessel, and consequent ECM production and intimal thickening, are the key events in the development of CAV (Figure 3B). The SMCs are thought to derive either from the proliferation of existing vascular SMCs, or from recruited circulating host cells [78]. In addition, the presence of TGFβ may induce endothelial-to-mesenchymal transition, during which ECs differentiate into cardiac SMC- and fibroblast-like cells [95]. The pathological smooth muscle cells are located in the deeper regions of the tunica intima next to the tunica media [96]. Different growth factors secreted by immune cells and CMs, trigger SMC proliferation, but these mechanisms are still incompletely understood [97]. SMCs contribute to the development of fibrosis seen in the tunica intima by increased collagen synthesis induced by the presence of increased TGFβ. In addition, the production of connective tissue growth factor (CTGF) by SMCs is upregulated, leading to increased ECM production and fibroblast proliferation. On the other hand BMP, a member of the TGFβ superfamily, may present anti-fibrotic effects on the lesion [98].

### 5.3. Immune Cells

Immune cells found in CAV lesions are mostly located in the intimal part subjacent to the luminal endothelium. NK cells and macrophages are commonly found in CAV lesions, but lymphocytes, presenting mainly T cells, are believed to be the immune cell drivers of CAV [78].

#### 5.3.1. T Cells

When encountering alloantigens presented by HLA molecules, effector CD8+ T cells may act via cytolysis and CD4+ T cells via secretion of cytokines, such as IFNγ and TGFβ [99]. In CAV, the targets of effector T cells are the ECs lining the vessel lumen expressing high levels of HLA molecules, whereas SMCs, which express only low levels of HLA are less often targeted. In addition to CD4+ and CD8+ T cells, also memory T cells, Th17 cells, regulatory T cells (Tregs) and γδ T cells are found in CAV lesions [97].

#### 5.3.2. B Cells

Fibrotic areas of the vasculature in CAV have B cell infiltrates, indicating that B cells contribute to CAV pathogenesis [100]. Firstly, they may differentiate into plasma cells, which produce DSAs and upon binding to the target antigens, activate the complement system. However, in 30–50% of all HTx patients with CAV, no detectable circulating antibodies are reported [101]. This implies that B cells have also other roles, such as stimulation of T cells.

#### 5.3.3. NK Cells

NK cells seem to be important mediators of CAV development. In fact, NK cell deficient mice develop only minor, if any, CAV [102]. NK cells may be activated via proinflammatory cytokine stimuli, but they can also recognize non-self similarly to cells of the adaptive immune system. Activated NK cells secrete cytokines such as IFNγ, which may promote SMC proliferation [78] and antigen-specific CD8+ T cell response [103].

#### 5.3.4. Macrophages

Macrophages, mast cells and lymphocytes secrete also other factors such as increased amounts of PDGF [104,105,106], FGF [107] and TGF, which stimulate SMC proliferation resulting in intimal hyperplasia and vascular remodeling. Upon stimulation by TGFβ, macrophages also produce matrix metalloproteinases (MMPs), which are involved in remodeling and activation of fibrosis [97]. Phagocytosis of dying cells may induce the anti-inflammatory (“M2”) phenotype in macrophages, which are increased in the neo-intima of CAV affected arteries [108]. Anti-inflammatory macrophages promote tissue growth, and enhance fibrotic process in CAV by proliferation and differentiation of fibroblasts and myofibroblasts.

#### 5.3.5. Mast Cells

Mast cells are increased at perivascular sites of occluded intramyocardial vessels and in the interstitium, and together with macrophages they are the main source of FGF contributing to enhanced inflammation, neovascularization, and fibrosis in a rat model of CAV [107].

### 5.4. Cardiomyocytes

CMs are spatially further away from the coronary artery intima, but studies show that VEGF secreted by CMs and mononuclear immune cells upon ischemia or alloimmune response triggers CAV formation [109]. The mechanism is suggested to take place either through macrophage stimulation or via inducing SMC migration to the site of inflammation.

## 6. Conclusions and Future Directions

In order to improve heart allograft survival after transplantation, precise understanding of the cellular and molecular mechanisms driving the pathological changes is critical. While much of the transplant research has focused on adaptive immune response, a growing body of research shows the importance of innate immune response and the cross-talk between immune and structural cells of the heart in transplantation biology. For example, the importance of the transplant endothelium in driving pathological changes observed in IRI, acute rejection and CAV has been well recognized. However, the endothelial cell subtypes which are altered after transplantation, or the cellular communication leading to these changes, remain to be characterized. Furthermore, there is very little knowledge on the role of the other structural cells of the heart, such as fibroblasts or CMs, in the development of allograft dysfunction and failure. Fibrosis may occur already at a very early stage after transplantation [110], and it is a cardinal late feature in CAV lesions, but the cellular heterogeneity and communication between immune cells and stromal cells such as pericytes, SMCs and different fibroblasts, responsible for transplant fibrosis, are not well understood.

Structural immunity is an emerging field of research, and it is becoming increasingly clear that structural cells of organs are essential partners of immune responses (Krausgruber 2020). Thus, an important future goal in mechanistic transplantation studies will be creating a communication network of structural and immune cells in the heart allograft. As pointed out in this review, single cell studies in normal heart and in cardiac ischemia have advanced our understanding of both the cellular composition of the heart and of the ischemia-induced changes in the structural cell composition and cell communication in the heart. Data from multi-omics studies utilizing spatial transcriptomics and single cell epigenomic, transcriptomic and proteomic data from transplanted hearts, will give us a novel approach to understanding the allograft mechanisms at a molecular and cellular level. Furthermore, this will permit the development of improved therapies for allograft protection.
